# Health Tract, No. 310

**Published:** 1867-11

**Authors:** 


### HEALTH TRACT, No. 310.

### THE DEAD WIFE.

There is no sorrow so crushing, so overwhelming, so utterly irremediable as that for the dear dead wife; the wife of your first love, of your buoyant, hopeful youth, with all its new experiences, its sweet revelations, its early struggles, its mutual aims, its hopes, its labors, and its fruitions; for long years together you worked side by side, hand in hand; she shared your troubles, and kissed away half their severity; she doubled your gladness by the pleasure which it gave her to see you happy; and when in the lapse of years you had arrived at a position to enable you to take life easy and enjoy it, as you had never done before, a heavenly hand takes her from your side and transports her into the presence of God, where you may not follow her now. You want to tell her how sweetly she died; how her friends gathered around her funeral bier and, in their affection, strewed white flowers upon her bosom; how lovingly and long they gazed on the dear familiar face, so beautifully calm in death;—a heavenly sweetness so pervading every lineament, as to give to it an angel seeming. You want to tell her, too, how the last, long, fond kiss almost broke your heart, and how you wanted to die when they covered her face from your sight forever; and then, as the weary weeks pass on, how busy memory brings up the forgotten past, with its long array of loving acts; of spontaneous tendernesses; of self-abnegations; of sleepless vigilence; of instinctive solicitude;—how you would give your life away for one short interview. But it cannot be; she is an angel now, and in her heavenly purity waits in patient affection for the time when it shall be the Master's will to bring you to her feet, and make of you an angel too. Beautifully has it been said ot

THE DEAD WIFE.

"In comparison with that loss, all other bereavements are trifles. The wife! she who fills so large a space in the domestic heaven; she who is so busy, so unweary—bitter, bitter is the tear that falls on her clay. You stand beside her grave and think of the past; an amber-colored pathway, where the sun shone upon beautiful flowers, or the stars hung glittering in the sky. No thorns are remembered above that sweet clay, save those your own hand may have unwittingly planted. Her noble, tender heart lies open to your inmost sight. You think of her as all gentleness, all beauty, and all purity. But she is dead! The dear head that so often laid upon your bosom, now rests upon a pillow of clay. The soft hands that ministered so untiringly to your every want are folded, white and cold, beneath the gloomy portals. The heart whose every beat measured an eternity of love, lies still under your feet. And there is no white arm over your shoulder now; no speaking face to look up in the eye of love; no smile to greet you at night-fall—and the clock ticks and strikes, and ticks again, it was sweet music when she could hear it; now it seems to knell only the hours through which you watched the shadows of death gathering upon her sweet face; but many a tale it telleth ot joys past, sorrows shared, and beautiful words and deeds registered above. You feel assured that she is in a happier world, and that with an angel presence she is often at your side. Cherish these emotions. Let her holy presence be as a charm to keep you from evil. Never forget what she has been to yon, and be tender of her memory!" But if desolations like these sweep resistlessly over the heart of the stout, strong man when his wife has been taken from his side, how incomputably greater must be the sorrow of the widowed bosom when he has been removed upon whose arm she so wholly leaned, upon whose counsel she so wholly rested; and upon whose providence she so implicitly relied. Give your warmest sympathies, then, to the widowed heart reader, and no longer wonder that Divinity has made it one of the evidences of fitness for the heavenly mansions, "To visit the widow," to stand by her, ever ready to lend her your countenance, your counsel, your aid, your support, and your defence.

